# Preparation and Biomedical Applications of Spherical Cellulose Hydrogels: A Mini-Review

**DOI:** 10.3390/gels12050349

**Published:** 2026-04-22

**Authors:** Kaiqing Yang, Juping Zheng, Shiquan Shen, Chao Li, Yuzhu Song, Yichen Tian

**Affiliations:** 1 Research Center of Molecular Medicine of Yunnan Province, Faculty of Life Science and Technology, Kunming University of Science and Technology, Kunming 650500, China; 2 Yunnan Provincial Key Laboratory of Tea Science, Tea Research Institute, Yunnan Academy of Agricultural Sciences, Kunming 650201, China

**Keywords:** spherical cellulose hydrogels, bottom-up approaches, fabrication processes, biomedical applications, controlled drug delivery, biomaterials

## Abstract

As the most abundant natural polymer on Earth, cellulose offers distinct advantages including renewability, biocompatibility, and modifiability. Among its various morphologies, spherical cellulose hydrogels (SCHs) represent a particularly versatile form ranging from micrometer to millimeter scales. They possess a unique hydrophilic 3D network, excellent flowability, high specific surface area, and outstanding mechanical stability, demonstrating great potential for biomedical applications. This mini-review highlights the primary bottom-up fabrication strategies for SCHs, including dripping, spraying, emulsion, and microfluidics, and the mechanisms by which different fabrication processes regulate their size, morphology, and structure are elucidated. On this basis, the recent advancements in SCHs across key biomedical domains, specifically in chromatographic separation, controlled drug delivery, tissue engineering, and wound healing, are discussed. Finally, the current challenges and future directions in this field are summarized and predicted, aiming to provide a reference for the development and application of high-performance cellulose-based biomaterials.

## 1. Introduction

As the most abundant bio-renewable material on Earth, cellulose is produced at an estimated annual rate of 1.5 × 10^12^ tons via photosynthesis in plants [1]. This exceptional abundance positions cellulose as a virtually inexhaustible feedstock to meet the growing global demand for environmentally benign and biocompatible products [2]. The low cost of cellulose, combined with its renewability, biocompatibility, robust mechanical properties, and suitability for facile surface chemical modification, renders it an ideal candidate for material development in biomedical applications [3]. Notably, the abundant hydroxyl groups distributed along cellulose molecular chains provide a versatile platform for chemical modification, giving rise to a diverse family of cellulose derivatives—including carboxymethyl cellulose (CMC) [4], methyl cellulose (MC) [5], and hydroxypropyl cellulose (HPC) [6]—each featuring distinct functionalities. By introducing functional groups, the water solubility, gelation behavior, and intermolecular interactions of cellulose can be systematically modulated, thus endowing tailored properties and establishing a versatile material basis for the subsequent design of functionally customized architectures [7].

Controlling the morphology and dimensions of cellulose-based materials has emerged as an active area of research. Conventionally employed in the paper and textile industries, cellulose is processed by casting and drying in molds or extrusion and drawing into fibers for textiles [8]. More recently, ball milling, high-pressure homogenization, acid hydrolysis, and enzymatic hydrolysis have enabled the downsizing of cellulose to nanoscale dimensions, yielding zero-dimensional (0D) or one-dimensional (1D) cellulosic nanomaterials, primarily including cellulose nanocrystals (CNCs), cellulose nanofibers (CNFs), and cellulose nanoparticles (CNPs) [9]. Beyond these nanoscale architectures, a variety of processing techniques—including solvent casting, freeze-drying, three-dimensional (3D) printing, and microfluidics—enable the fabrication of two-dimensional (2D) and 3D cellulose-based materials such as hydrogels, aerogels, scaffolds, sponges, and films [10]. Cellulose can also serve as a matrix for the preparation of spherical hydrogels with diameters tunable from micrometers to millimeters.

Spherical cellulose hydrogels (SCHs) represent micrometer-to-millimeter-scale spherical cellulose materials featuring a hydrophilic 3D network structure. Owing to their high water content, tunable properties, and structural similarity to the native extracellular matrix (ECM), these materials have been employed as substrates for cell culture, scaffolds for tissue engineering, and carriers for drug and protein delivery [11]. Compared to their fibrous or film counterparts, spherical geometries offer superior flowability, higher specific surface area, more uniform stress distribution, and excellent mechanical stability. More importantly, by virtue of their abundant hydroxyl groups and unique hydrophilic 3D network architecture, cellulose hydrogels are endowed with exceptional biomimetic characteristics and remarkable potential for functional integration [12]. These exceptional attributes have translated into widespread commercial adoption [13,14,15,16]. For instance, cellulose spheres with diameters ranging from 3 μm to 4 mm have been developed by Rengo Co., Ltd. (Tokyo, Japan) and are used as carriers, cosmetic raw materials, and abrasives, among others. These cellulose spheres are biodegradable not only in soil but also in seawater, having obtained the OK biodegradable MARINE certification, making them a sustainable alternative to plastic microbeads. In addition, porous variants (e.g., 2 mm and 4 mm) can achieve long-term controlled release of active substances through embedding technology [14]. Similarly, cellulose microspheres with diameters of 40 to 130 μm have been commercialized by JNC Corporation (Tokyo, Japan) for use as chromatography media in the purification of proteins, enzymes, and other biomolecules [15]. Likewise, a range of cellulose-based ion-exchange media for the adsorptive separation of cations, anions, and other specific targets are commercially available from Cytiva (Marlborough, MA, USA) [17]. Collectively, these commercial examples underscore the translational potential of this material class—from laboratory research to practical application.

Given the frequent lack of comprehensiveness and timeliness in existing reviews, few have systematically addressed SCHs as a distinct morphology, compared different preparation methods in terms of particle size, yield, sphericity, and scale-up feasibility, or offered in-depth analysis of their translational potential. To fill these gaps, this review focuses on SCHs, specifically millimeter-scale beads and micrometer-scale microspheres that possess a hydrophilic 3D network architecture. It systematically surveys recent advances in fabrication strategies for this field and comprehensively summarizes their current applications in key biomedical fields—including drug delivery, tissue engineering, and chromatographic separation. We also cover emerging areas such as wound healing and hemostasis, commercial SCH products, industrial scale-up trade-offs among fabrication methods, and key barriers to clinical translation.

## 2. Preparation Strategies for Spherical Cellulose Materials

**Figure 1:**
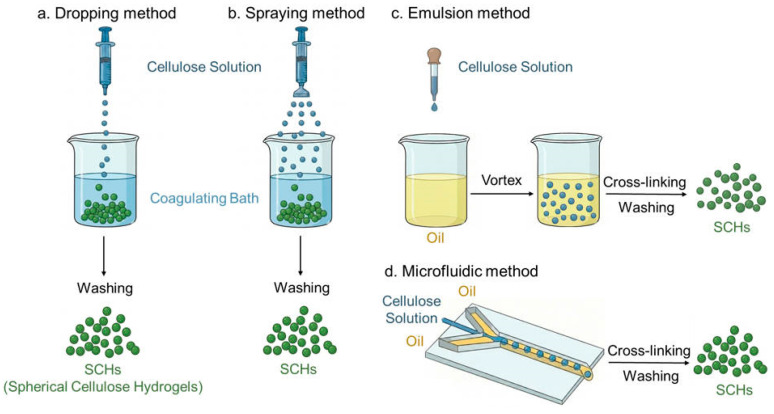
Schematic diagram of the preparation of spherical cellulose based on dissolution-regeneration techniques: (**a**) dropping, (**b**) spraying, (**c**) emulsion and (**d**) microfluidic methods.

SCHs are typically fabricated via bottom-up approaches [18], in contrast to top-down strategies such as mechanical shearing and acid hydrolysis. While such top-down methods can disintegrate native cellulose into nanocellulose while retaining its intrinsic crystalline structure and high mechanical strength, they afford only limited control over particle morphology and dimensions [19]. The core principle underpinning the bottom-up strategy involves disrupting the crystalline network of native cellulose through dissolution, followed by regeneration to restructure the polymer into spherical architectures with a three-dimensional network, thus allowing for precise control over morphology and structural design [20]. The process typically comprises three sequential steps: (1) dissolving cellulose or its derivatives in a suitable solvent; (2) converting the resulting solution into spherical droplets via dedicated techniques; and (3) solidifying or regenerating these droplets to form SCHs. Common fabrication techniques include the dripping, spraying, emulsion, and microfluidic methods (Figure 1).

### 2.1. Dripping Method

The dripping method involves extruding a viscous cellulose solution through a syringe nozzle, where the extruded stream contracts into spherical droplets under surface tension before falling into a coagulation bath (Figure 1a). Cellulose spheres fabricated via the dripping method typically exhibit diameters ranging from 0.5 to 3 mm, with the uniformity of their spherical morphology governed by parameters such as dripping height, extrusion rate, and the viscosity of the cellulose solution. The preparation of spherical cellulose beads was pioneered in a 1951 patent, where the method involved simply dropping a viscous cellulose solution directly into a coagulation bath [21]. Cellulose (3 wt.%) was dissolved in a LiCl/DMAc solvent system and subsequently regenerated into spherical particles with diameters of 1.7–1.8 mm via dropwise addition into ethanol by Li et al. [22]. Although the dripping method is limited by low production efficiency, which restricts its scalability and means that commercial products manufactured via this method remain limited, its operational simplicity and minimal equipment requirements render it valuable for laboratory-scale research and the preparation of specific large-diameter beads.

### 2.2. Spraying Method

To achieve smaller droplet sizes, modifications to the injection system (e.g., pneumatic assistance or rotary shear) can be implemented (Figure 1b). In the spraying method, for instance, the cellulose solution and air enter the spray nozzle through two separate inlets, where they are sheared under high pressure into micron-sized droplets that are subsequently solidified into microspheres. As a derivation of the dripping method, this approach modifies the injection system to achieve finer droplet formation; consequently, compared to the conventional dripping method, the microspheres fabricated via the spraying method are smaller, feature higher size uniformity, and are more amenable to automated mass production [23]. Jacquart et al. fabricated carboxymethyl cellulose microspheres via spray drying, demonstrating that microsphere size and porosity could be readily tuned by varying the cellulose concentration. In the concentration range of 2–10 g/L, higher cellulose concentrations consistently produced larger microspheres [24]. Furthermore, the application of high voltage electricity to the nozzle imparts electrical charges to the cellulose solution ejected from the spray head. Owing to electrostatic repulsion, the as-prepared microspheres exhibit excellent monodispersity [25]. Monodisperse cellulose microspheres were fabricated by Du et al. via electrospraying, with their size tunable from 200 to 1800 μm by varying the nozzle diameter and applied voltage [26]. Commercially, Rengo Co., Ltd. produces micro-sized cellulose spheres via mechanical granulation (e.g., Viscopearl P), a process closely related to spraying-based techniques, for use as cosmetic raw materials, abrasives, and ink matting agents [14].

### 2.3. Emulsion Method

The emulsion method is regarded as the gold standard for fabricating cellulose microspheres [27], capable of producing both micrometer-scale and nanometer-scale particles with the advantages of simple processing, good reproducibility, and scalability. The principle involves dispersing a cellulose solution uniformly in an organic solvent under shear forces—such as high-pressure homogenization, mechanical stirring, or ultrasonication—assisted by a surfactant to form an emulsion [28]. Subsequent regeneration via acid treatment or exposure to a non-solvent for cellulose (e.g., ethanol or acetone) solidifies the cellulose droplets into microspheres (Figure 1c). The diameter of microspheres prepared via the emulsion method is governed by multiple factors, including shear rate, the type and concentration of surfactant, the nature of the dispersing medium, and the viscosity of the cellulose solution [29]. For instance, higher shear rates yield smaller microspheres [30,31]. Luo et al. successfully fabricated cellulose microspheres with average diameters of 5–1000 μm using a NaOH/urea aqueous system to dissolve cellulose (as the aqueous phase), with the size tunability achieved by adjusting experimental parameters such as surfactant concentration, aqueous-to-oil phase ratio, and stirring rate [32]. Xu et al. dissolved pulp cellulose in a tetraethylammonium hydroxide (TEAH)/urea solvent system and fabricated porous cellulose microspheres through a simple process of emulsification, acid coagulation, and oven drying. By varying the concentration of the surfactant Span 80, the microsphere size could be tuned within the range of 20–224 μm, with pore volumes ranging from 8.24 to 10.20 mL/g [33].

Microspheres with tailored properties can be fabricated via emulsion methods involving cellulose solution modification, thereby expanding their potential applications [11]. For instance, microspheres with specific functionalities can be prepared by incorporating functional additives into the cellulose solution. Liu et al., for example, incorporated Fe_3_O_4_ nanoparticles into a cellulose solution prior to emulsification, yielding magnetic cellulose microspheres that were subsequently applied to the rapid screening of enzyme inhibitors [34]. Simões et al. encapsulated eugenol within a cellulose derivative solution to achieve sustained drug release [35]. In contrast to the dripping method, the emulsion method enables the production of highly reproducible microspheres without requiring specialized large-scale equipment, a combination of advantages that has facilitated the commercial production of emulsion-derived cellulose microspheres. The emulsion method, however, is not without limitations. The resulting microspheres often exhibit a broad size distribution, necessitating subsequent sieving to obtain uniformly sized particles. Furthermore, the process involves the use of large quantities of organic solvents, which tend to adhere to the microsphere surfaces and are difficult to remove completely.

### 2.4. Microfluidic Method

Microfluidic technology—a versatile chip-based platform—enables precise manipulation of multiphase flows [36]. Similar to the emulsion method, microfluidics leverages the principle of liquid–liquid immiscibility to generate cellulose droplets with precisely controlled size, shape, and morphology [37]. During fabrication, the cellulose solution (dispersed phase) and the continuous phase (typically an organic solvent or other immiscible liquid) are co-introduced into a microfluidic channel. Under shear forces exerted by the continuous phase, the cellulose solution is sheared into uniformly sized microdroplets, which are subsequently solidified into microspheres upon entering a coagulation bath (Figure 1d). For instance, Zhang et al. employed microfluidics to fabricate cellulose acetate microspheres with tunable porosity and size. The microsphere diameter was found to correlate with the continuous-phase flow rate: increasing the flow rate from 100 to 400 μL/min reduced the diameter by a factor of three [38]. This fabrication strategy yields microspheres with a narrow size distribution and high monodispersity—a characteristic that avoids erratic drug release profiles and mitigates the risk of off-target accumulation in vivo, making them particularly promising for biopharmaceutical applications [39]. However, its scalability for industrial production is limited by the inherently low throughput of the microfluidic systems, and large-scale commercial production has not yet been realized.

In summary, each method offers distinct advantages (e.g., high sphericity for dripping, scalability for spraying and emulsion, excellent monodispersity for microfluidics) and limitations (e.g., low yield, broad size distribution, or low throughput), so the selection of an appropriate preparation strategy should be based on the specific requirements of the intended biomedical application. However, from an industrial scale-up perspective, the existing methods each prioritize different trade-offs among cost, throughput, monodispersity, safety, and mechanical robustness, and no single method currently satisfies all these requirements simultaneously. Take the emulsion method as an example: the particle size distribution is highly sensitive to stirring speed, surfactant concentration, and temperature, which easily leads to batch-to-batch variations [40]. Moreover, the large amounts of organic solvents required are difficult to completely remove from the final microspheres, making their residual presence problematic for biomedical applications. Microfluidics, on the other hand, offers excellent monodispersity but suffers from low throughput, and ton-scale production would remain impractical until parallelization technology matures [41,42]. Spraying and emulsion methods are relatively more mature for industrial use, yet both still require rigorous post-processing to ensure solvent removal and sterility [26,43]. To facilitate a direct comparison of the fabrication methods outlined above, their key characteristics—including attainable size range, throughput, sphericity, and core advantages—are systematically summarized in Table 1. Notably, the porosity and mechanical strength of SCHs are strongly dependent on cellulose solution concentration, which differs considerably across various preparation methods [44]. In general, higher cellulose concentrations give rise to improved mechanical strength but reduced porosity [45]. As direct comparative studies that systematically elucidate the effects of different preparation techniques on these two properties remain lacking, these data are not included in Table 1.

## 3. Biomedical Applications of Spherical Cellulose Materials

**Figure 2:**
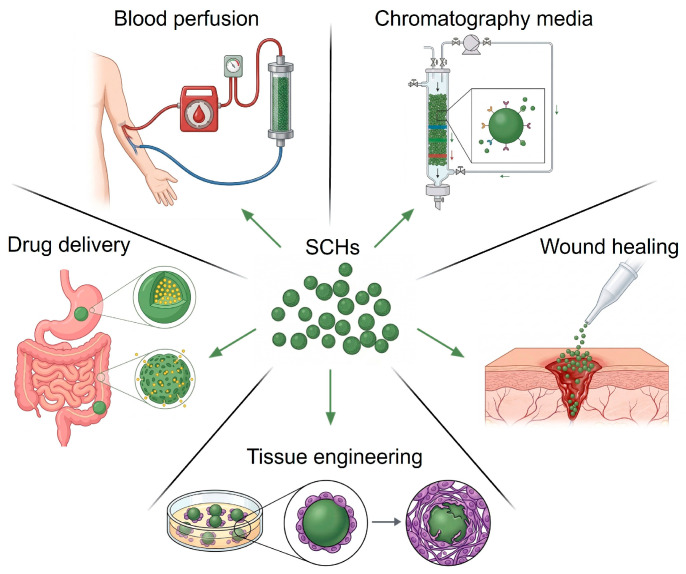
Schematic representation of the diverse biomedical applications of spherical cellulose hydrogels (SCHs).

Spherical materials exhibit distinct advantages—including superior flowability, high specific surface area, homogeneous stress distribution, and outstanding mechanical stability—that make them highly valuable in biomedical applications [46,47]. As a matrix material, cellulose serves to further amplify and extend these inherent merits. Their favorable flowability enables uniform mixing and dispersion, as well as rapid entry into the bloodstream following administration. A high specific surface area provides abundant active sites and facilitates more uniform functionalization with other chemical groups, making them well-suited for biosensing and diagnostic applications. Furthermore, their homogeneous stress distribution and excellent mechanical stability ensure structural integrity under mechanical stress, rendering them resistant to fracture. Consequently, cellulose microspheres have found widespread application across a broad spectrum of biomedical fields, including (but not limited to) chromatography, separation technology, adsorbents, biocatalyst immobilization, and as carrier systems [48,49,50]. As illustrated in Figure 2, SCHs provide a highly versatile platform across diverse clinical and research domains. The following sections will systematically review the applications of SCHs in chromatographic purification, drug delivery, tissue engineering, and other related fields.

### 3.1. Chromatography Media

Chromatography is a fundamental separation and analytical technique. Liquid chromatography (LC) has found widespread application in biomedical fields owing to its high resolution and rapid separation speed [51]. The uniform particle size distribution of spherical materials ensures the homogeneity and stability of chromatographic columns, thereby enhancing separation efficiency. Cellulose microspheres are extensively used as stationary phases for the adsorption or purification of specific substances in chromatographic analysis. This broad utility stems from their excellent biosafety, inherent hydrophilicity, superior mechanical strength relative to other polysaccharide-based materials (e.g., agarose) [52], and weak non-specific adsorption.

The adsorption and purification efficiency of cellulose microspheres is primarily determined by two key parameters: pore size and the nature of the functional groups grafted onto their surface [23]. An appropriate pore size enhances adsorption capacity, while surface functionalization with different chemical groups enables selective capture of specific target molecules. For instance, Qiao et al. fabricated porous cellulose microspheres via the thermal removal of agarose from pre-formed agarose/cellulose composite microspheres. The resulting structures exhibited a specific surface area of 173.85 m^2^/g and a pore size range of 0.5–1.0 μm. Following modification with diethylaminoethyl chloride (DEAE-HCl), the microspheres achieved a maximum adsorption capacity of 409.46 mg/g for bovine serum albumin (BSA). This superior adsorption performance—attributed to the interconnected macroporous architecture that substantially reduces mass transfer resistance and enhances adsorption kinetics—far exceeded that of non-porous control groups [53]. Wang et al. fabricated cellulose microspheres with carbon nanotube (CNT)-tuned pore sizes, achieving a pore diameter of 2.69 ± 0.57 μm and a high specific surface area of 147.47 m^2^/g. Following functionalization with quaternary ammonium groups, the microspheres were employed as anion adsorbents for the adsorption of pancreatic kininogenase (PK). Although cellulose exhibits improved mechanical strength compared to agarose, inadequate mechanical robustness is recognized as the primary challenge for cellulose-based chromatography media [54]. Zhao et al., for example, prepared porous cellulose–agarose microspheres with high specific surface area and enhanced mechanical strength via chemical cross-linking for the high-affinity adsorption of bovine hemoglobin. In their approach, the cross-linking agent was mixed with the cellulose and agarose solution, whereby the hydrogen-bonding network within cellulose was replaced by a covalently cross-linked architecture, leading to improved mechanical stability of the regenerated microspheres [55].

### 3.2. Drug Delivery

Drug delivery systems play a pivotal role in modern therapeutics, with the central objective of maintaining therapeutic drug concentrations for adequate durations in vivo [56]. Excessively high drug concentrations may induce toxicity, whereas suboptimal concentrations or insufficient duration of action can compromise therapeutic efficacy. Sustained drug release is one of the key advantages of cellulose-based carriers in drug delivery systems. Cellulose enables controlled release through multiple mechanisms—including water retention, film formation, enhanced adhesion, and rheological modulation. Moreover, its high safety profile, stability, and biocompatibility position it as an ideal carrier for therapeutic delivery [8].

Administration routes for cellulose-based drug delivery materials encompass oral, ocular, tumor-targeted, and transdermal delivery, among others [57]. The release mechanisms typically operate in response to stimuli such as light, pH, or temperature. Gong et al., for instance, designed pH-responsive carboxylated cellulose microspheres for the oral delivery of insulin. Insulin was loaded into the cellulose microspheres via electrostatic interactions, and its release within the intestine was governed by the ionization and proton balance of the carboxyl groups [58]. Zhang et al., for example, fabricated bio-inspired smart microcapsules assembled from CNFs. These microcapsules feature an open network-like shell that can be utilized for drug delivery. With their tunable nanostructure and pore size, the microcapsule shells undergo intelligent opening and closing in response to pH changes, enabling the controlled release of entrapped silver ions [59]. Yang et al. prepared thermoresponsive composite microspheres based on CNCs. These microspheres, characterized by their controllable size and high encapsulation efficiency, are capable of tailoring drug release in response to variations in physiological temperature [60]. Smart hydrogel wound dressing engineered by Li et al. exhibited stimuli-responsive sequential release, delivering only 33.24% of epicatechin within 24 h while achieving complete eradication of *S. aureus* and *E. coli*, thereby enabling sustained antibacterial and anti-inflammatory therapy for infected wounds [61]. Myrick et al. fabricated cellulose hydrogel microspheres based on cellulose ether and hydroxypropyl cellulose that are dual-responsive to both temperature and pH for the encapsulation of glucose. In vivo release kinetics measurements revealed that, compared to control groups, the hydrogel microspheres enabled significantly higher glucose concentrations to be maintained in plasma over an extended period [62]. These representative examples illustrate the versatility of SCHs in drug delivery applications, and their key characteristics are summarized in Table 2.

### 3.3. Tissue Engineering

Cells, as the fundamental units of tissues and living organisms, rely on cell culture techniques that possess enormous application potential across diverse fields, including drug screening, cancer therapy, stem cell research, and tissue engineering [63]. In contrast to conventional 2D cell culture, 3D cell culture offers a larger contact area between cells and promotes enhanced interactions between cells and the ECM, thereby better mimicking the native in vivo microenvironment of tissues [64]. Hydrogel microspheres not only simulate the highly hydrated nature of the extracellular matrix but also offer abundant space for cell growth and migration through their unique 3D porous architecture. Due to their high porosity, suitable mechanical strength, and excellent biocompatibility, cellulose-based materials have emerged as a mainstream choice for 3D cell culture [65].

Commercial cellulose-based microparticles specifically designed for cell culture are already on the market. For instance, Cytopore^TM^ offers macroporous cellulose spherical microcarriers with diameters ranging from 200 to 280 μm, composed entirely of cotton cellulose [16]. These microcarriers suit stirred-tank bioreactors and support high-density cell culture. Their macroporous structure allows cells to grow in the internal 3D space, thereby significantly boosting cell yield. In a practical application, Wang et al. fabricated 450 μm porous cellulose-based microspheres from bacterial cellulose-chitosan composites and successfully used them in a rat model for knee cartilage repair—they not only caused no immune complications but also significantly promoted cartilage regeneration [66].

Furthermore, cellulose-based microspheres have demonstrated significant potential in the field of skin tissue regeneration [67]. For instance, hollow bacterial cellulose microspheres fabricated to promote cell proliferation have been shown to accelerate wound healing in a rat skin model [68]. In another study, Xu et al. encapsulated human adipose-derived stem cells (ASCs) in a chitosan-cellulose hydrogel with embedded drug-loaded microspheres. The encapsulated cells maintained >98% viability over 14 days, and the system showed sustained antibacterial activity, highlighting its potential for cell/drug delivery in wound healing [69].

### 3.4. Other Applications

Owing to their unique structural features and high specific surface area, spherical cellulose materials exhibit promising application potential in diverse fields. Beyond the aforementioned mainstream applications, spherical cellulose materials also show outstanding performance in areas such as hemostatic materials [70,71] and blood purification [72,73]. Xu et al. engineered cellulose microspheres with a wrinkled surface morphology. In conjunction with their hydrophilic nanoporous 3D network, these microspheres enable hemostasis of acute non-compressible hemorrhage within seconds [71]. Tian et al. and Tang et al. employed carboxymethyl cellulose hydrogel beads and hydrophobically modified cellulose microspheres, respectively, for hemoperfusion to remove low-density lipoprotein (LDL) and bilirubin in blood [73,74]. These emerging applications further underscore the practical value and translational potential of SCHs in critical healthcare scenarios.

## 4. Conclusions and Perspectives

In recent years, spherical cellulose materials have gradually replaced conventional petroleum-based plastics, such as polystyrene and polyethylene microspheres, thanks to their renewable, biodegradable, and eco-friendly nature. Featuring high specific surface area, outstanding uniformity, excellent hydrophilicity, and biocompatibility, spherical cellulose materials show extraordinary potential for a wide spectrum of biomedical applications. However, their intrinsically poor solubility has long limited their precise processing and broader application. Recently, the challenges associated with cellulose dissolution and processing have been effectively relieved by the development of advanced green solvent systems and the rational use of cellulose derivatives (e.g., carboxymethyl cellulose and cellulose acetate) [75]. These advances have laid a solid foundation for the controllable synthesis of SCHs. Among current fabrication strategies, traditional methods including dripping, and emulsions remain the most widely used for SCH production, owing to their simplicity and cost efficiency. Meanwhile, emerging techniques such as microfluidics and electrospraying are propelling SCH development toward precise control over particle size and structure. The persistent demand for innovative and efficient therapeutic strategies in drug delivery, cell culture, and tissue engineering has spurred rapid growth in biomedical particulate materials.

Despite their excellent in vitro performance, the clinical translation of SCHs faces several formidable barriers. Many current biomedical SCHs are designed as multi-component or modular platforms. While these designs offer precise therapeutic functions, validating their synergistic safety, long-term degradation pathways, and potential immunogenicity in complex, dynamic human physiological environments remains a significant translational hurdle. From a regulatory perspective, the transition from laboratory-grade to clinical-grade cellulose requires standardized purification protocols to eliminate potential pyrogens or endotoxins. Furthermore, while cellulose is generally recognized as safe, the introduction of novel chemical cross-linkers or functional grafts necessitates comprehensive long-term toxicity and degradation studies to satisfy the requirements of regulatory bodies. Establishing unified manufacturing standards for SCH materials will be a prerequisite for their inclusion in future pharmacopeias.

With the continuous innovation and optimization of novel fabrication technologies, SCHs are expected to be manufactured with higher efficiency and lower cost, and set to broaden their applications across diverse fields including medicine, biology, environmental science, electronics, and energy.

## Figures and Tables

**Table 1:** Comparison of different fabrication strategies for spherical cellulose hydrogels (SCHs).

Preparation Technique	Particle Size Range	Production Rate	Sphericity	Advantage
Dropping	0.5–4 mm	**	***	Ultra-simple operation, high sphericity of resultant microspheres
Spraying	1–100 μm	****	***	Suitable for large-scale powder production
Microfluidics	10–500 μm	*	****	Perfect monodispersity, customizable structure
Emulsion	10 μm–1 mm	***	***	Preparation process is simple, has good reproducibility and can be prepared on a large scale

Notes: ****: High; ***: Medium; **: Low; *: Extremely low. The ratings for production rate and sphericity are qualitative and relative comparisons based on the literature reviewed, rather than absolute or quantitatively defined thresholds. Different criteria were applied for each property according to the reported performance of each technique.

**Table 2:** Summary of key information on representative drug delivery studies.

Type of Cellulose	Delivery System/Formulation	Incorporated Drug	Stimuli Responsiveness	Study Level	Reference
Carboxylated cellulose microspheres (CCMs)	Microspheres	Insulin	pH	In vitro	[58]
Cellulose nanofibrils (CNFs)	Smart microcages	Silver nanoparticles, silicate nanoplatelets	pH	In vitro	[59]
Cellulose nanocrystals (CNCs)	Thermo-sensitive composite microspheres	Hydrophilic model drug (tetracycline hydrochloride)	Temperature	In vitro	[60]
Litchi husk cellulose (esterified)	Hydrogel with drug-loaded cellulose microspheres	G-AgNPs (green-synthesized silver nanoparticles), epicatechin	Sequential release	In vitro & in vivo	[61]
Hydroxypropyl cellulose (HPC)	Microgel dispersions	Glucose (dextrose)	Temperature & pH	In vitro and in vivo	[62]

## Data Availability

No new data were created or analyzed in this study.
